# Biotransformed Soybean Extract (BSE) Inhibits Melanoma Cell Growth and Viability *In Vitro*: Involvement of Nuclear Factor-Kappa B Signaling

**DOI:** 10.1371/journal.pone.0103248

**Published:** 2014-07-29

**Authors:** Fernanda Maria Pinto Vilela, Deeba N. Syed, Jean Christopher Chamcheu, Laura A. Calvo-Castro, Vanessa Silveira Fortes, Maria José Vieira Fonseca, Hasan Mukhtar

**Affiliations:** 1 Faculty of Pharmaceutical Sciences of Ribeirão Preto, University of São Paulo, Ribeirão Preto, Brazil; 2 Department of Dermatology, University of Wisconsin, Madison, Wisconsin, United States of America; 3 Centro de Investigación en Biotecnología, Instituto Tecnológico de Costa Rica, Cartago, Costa Rica; UCSF/VA Medical Center, United States of America

## Abstract

Melanoma is recognized as one of the most aggressive cancers with a relatively high propensity for metastasis. The prognosis of melanoma remains poor in spite of treatment advances, emphasizing the importance of additional preventive measures. Isoflavonoids have become not only potential chemopreventive, but also important therapeutic natural agents. We evaluated the antiproliferative and proapoptotic properties of biotransformed soybean extract (BSE) in A375 melanoma cells. Previous analyses demonstrated that the concentration of daidzein, genistein and aminoacids/peptides present in BSE, fermented by *Aspergillus awamori* is much higher than in the non biotransformed extract (NBSE). Experiments comparing the efficacy of the extracts in preventing cancer cell growth showed that treatment (24 h) of aggressive melanoma cells (A375 and 451Lu) with BSE resulted in a dose-dependent inhibition of growth and viability. In contrast, treatment with similar doses of NBSE failed to inhibit melanoma cell viability. Further studies in A375 cells showed that decrease in cell viability with BSE treatment (1.5–1.9 mg/ml; 24 h) was associated with induction of apoptosis. Immunoblot analysis revealed that BSE treatment resulted in induction of PARP cleavage, activation of caspase-3, -7, and -8 and increased expression of TRAIL and its receptor DR4. BSE did not activate the intrinsic apoptotic pathway in A375 cells, as no change was observed in caspase-9 expression. The expression of Bcl-2 apoptotic proteins such as Bid and Bax remained unaffected with BSE treated cells. Interestingly, we also showed that BSE treatment increased the phosphorylation and activation of IKK, IκBα degradation and p65/NF-κB translocation to the nucleus, and that stimulation of the NF-???B pathway was required for BSE-induced apoptosis of A375 cells. Our findings indicate that the biotransformation of soybean plays a crucial role in the extract anti-cancer effect observed in melanoma cells. However, further studies are warranted to define the active anti-cancer agent(s) present in BSE.

## Introduction

The incidence of cutaneous melanoma, a cancer of epidermal melanocytes continues to rise amongst the caucasian population [1]. Melanoma is characterized by an increased capacity to metastasize and, till date no suitable therapy for metastasized melanoma exists. In addition, resistance to apoptosis is considered to be a critical factor for therapy resistance [2], [3]. In recent years, the intake or treatment of skin with botanical antioxidants has served as a useful strategy for the prevention of skin damages [4]–[6] suggesting that pharmacological and nutraceutical agents that are mechanistically linked to inhibiting events in melanoma carcinogenesis are potential candidates for the prevention and treatment of this disease [7].

Soybean isoflavones are an interesting group of phytochemicals, shown to possess anti-cancer effects, including growth inhibition, cell cycle arrest, and induction of cell differentiation [8]. Soybean contains mainly isoflavone glycosides, such as daidzin and genistin, which upon being biotransformed into their aglycone forms, daidzein and genistein, become readily active, with greater bioavailability than the highly polar conjugated compounds [9], [10]. Thus, the enzymatic hydrolysis of phenolic glycosides using solid-state bioprocessing of soybean with food-grade fungi has been developed as a strategy to increase the concentration of free polyphenols and enhance the biological activity of soybean products [9], [11], [12].

Epidemiologic evidence, together with data from animal and *in vitro* studies, strongly supports a relationship between isoflavones and a lower risk of carcinogenesis [13]. In this context, inhibition of NF-κB signaling in tumor formation has been a major focus of study as a target of polyphenols and other natural and synthetic compounds [14]. Inactive NF-κB, comprising of p65/RelA and p50/p105 subunits, resides in the cytoplasm by remaining in a complex with its inhibitory unit, IκBα. In response to a variety of stimuli, the enzyme IKK phosphorylates IκBα, resulting in dissociation of IκBα from NF-κB, which then translocates to the nucleus, mediating a signal for cell survival [15]. However, it has also been shown that activation of the NF-κB pathway can exert a protective effect by inducing apoptosis of cancer cells, suggesting an additional role of NF-κB signaling in deciding cell fate [16].

In the present study, we investigated the effect of the biotransformed soybean extract (BSE) in 451Lu and A375 melanoma cells. This extract was prepared using solid-state biofermentation of soybean using *A. awamori* fungi as a β-glucosidase producer. The effect of BSE treatment in melanoma cells was compared with the effect of the non-biotransformed soybean extract (NBSE). The biotransformation process conferred, respectively, an approximate 50 and 42 fold higher contents of the soybean isoflavones daidzein and genistein to BSE, and much higher amounts of proteins and aminoacids/peptides when compared to NBSE (unpublished data). Also, examination of the cell death mechanism induced by BSE in melanoma cells identified the activation of NF-κB signaling as the primary event in BSE-induced apoptosis of melanoma cells.

## Results

### BSE decreased the viability of 451Lu and A375 human melanoma cells

**Figure 1:**
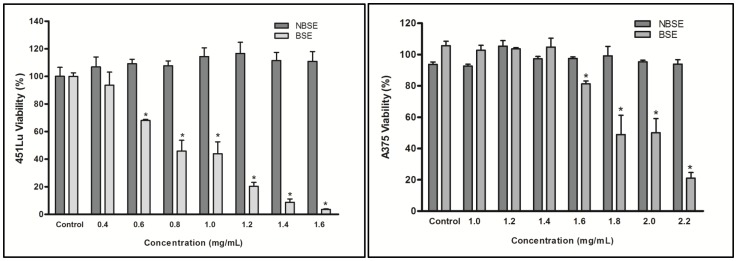
BSE and NBSE effect on melanoma cells growth and viability. 451Lu and A375 cell lines were treated with NBSE and BSE for 24(DMSO)-treated cells were regarded as 100% viable. Data is represented as mean ± SE of 3 independent experiments. *p<0.05 versus NBSE.

We first investigated the dose-dependent effect of BSE and NBSE treatment on the growth of 451Lu and A375 human melanoma cells using the MTT assay. BSE treatment of 451Lu (0–1.6 mg/mL) and A375 (0–2.2 mg/mL) melanoma cells for 24 h displayed a dose dependent decrease in cell growth/viability (Figure 1). The IC50 of BSE was estimated to be 0.8 mg/mL for 451LU and 1.7 mg/mL for A375 at 24 h, while NBSE treatment in both cell lines had no effect (in comparison to the untreated control) on cell viability at these doses. These data led to the selection of 1.5 to 1.9 mg/mL BSE doses for further mechanistic studies in the highly aggressive A375 melanoma cell line.

### BSE induced apoptosis in A375 melanoma cells

**Figure 2:**
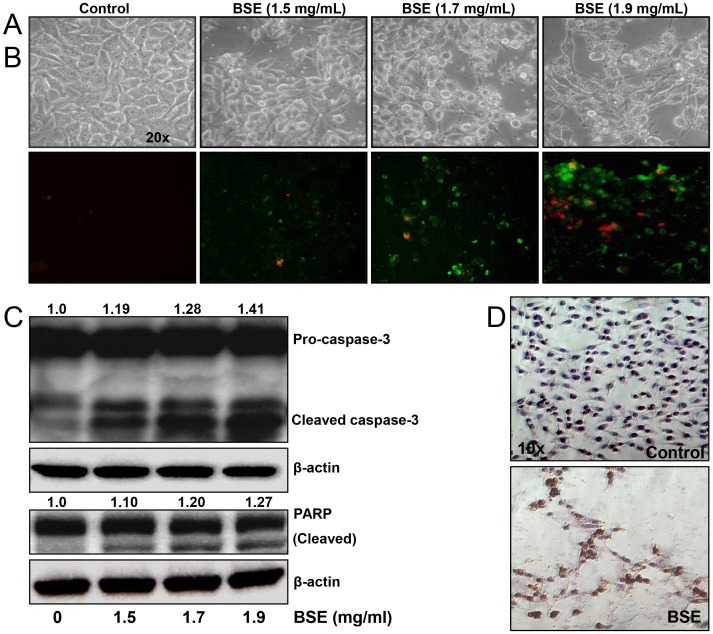
BSE treatment induced apoptosis in A375 melanoma cells. (A) Phase contrast microscopy: representative pictures of A375 cells treated with BSE (0–1.9 mg/mL) for 24 h. (B) Effect of BSE treatment (0–1.9 mg/mL) on apoptosis in A375 cells as demonstrated by annexin-V staining (green fluorescence). (C) Effect of BSE treatment (0–1.9 mg/mL) on activation of caspase-3 and PARP cleavage. Whole cell lysates were analyzed by immunoblot analysis. Equal loading was confirmed by reprobing for β-actin. The values above the figures represent relative density of the bands (cleaved caspase 3 and PARP) normalized to β-actin. Data shown are representative of three independent experiments. (D) Effect of BSE (1.7 mg/mL) treatment on the activation of caspase-3 as detected by immunocytochemistry. A representative picture from three independent experiments with similar results is shown.

A375 cells treated with BSE (1.5–1.9 mg/mL; 24 h) showed dose-dependent alterations in cell morphology (Figure 2A) and induction of apoptosis which was evident from the enhancement in fluorescent green Annexin V staining (Figure 2B). To further assess the molecular mechanism involved in BSE-induced apoptosis, we examined whether BSE treatment would result in activation of caspase-3 and PARP cleavage, which are considered important indicators of apoptotic cell death [17], [18]. Accordingly western blot analysis and immunocytochemistry showed that BSE treatment resulted in an increase in both caspase-3 activation (Figure 2C and D) and PARP cleavage (Figure 2C).

### BSE treatment did not result in activation of the intrinsic pathway in A375 melanoma cells

**Figure 3:**
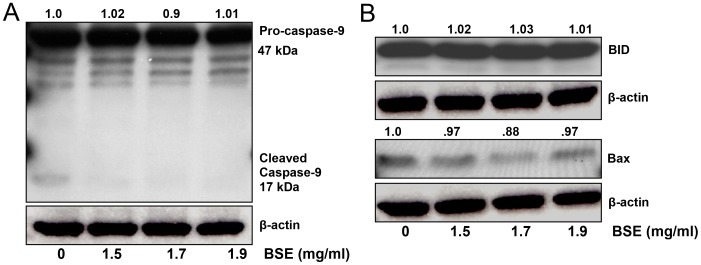
BSE treatment did not activate the intrinsic apoptotic pathway. (A) BSE treatment does not induce activation of caspase-9 in A375 melanoma cells. (B) BSE treatment does not up-regulate the expression of pro-apoptotic Bid and Bax proteins involved in the intrinsic apoptotic pathway. Whole cell lysates were analyzed by immunoblot analysis. Equal loading was confirmed by reprobing for β-actin. The values above the figures represent relative density of the bands normalized to β-actin. Data shown are representative of three independent experiments. A representative picture from three independent experiments with similar results is shown.

Apoptosis can be triggered by two major pathways: the extrinsic or death receptor pathway and the intrinsic or mitochondrial pathway [18]. We investigated if the intrinsic pathway was involved in BSE-mediated apoptosis of A375 melanoma cells. Interestingly, in comparison to the untreated control, caspase-9 activity was found to be unaffected by BSE treatment (Figure 3A). Furthermore, as shown in Figure 3b, protein expression of Bid and Bax, proapoptotic members of the Bcl-2 family, also remained unchanged in the BSE treated A375 melanoma cells.

### BSE treatment induced activation of the extrinsic pathway in A375 melanoma cells

**Figure 4:**
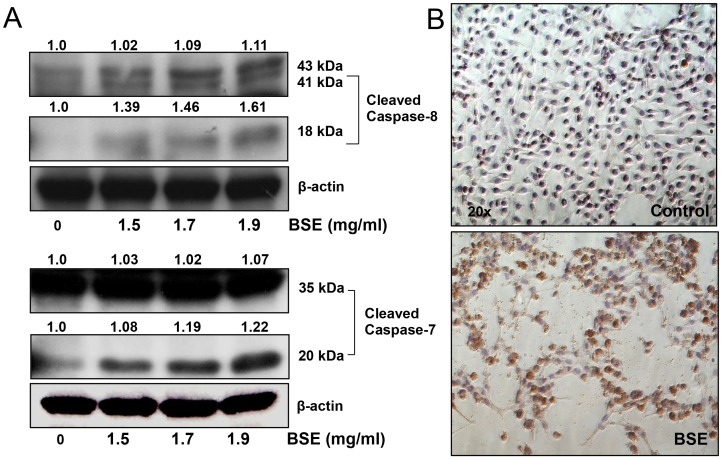
BSE treatment activated the extrinsic apoptotic pathway. (A) BSE treatment (24 h) induces activation of caspase-8 and caspase-7 as determined by immunoblot analysis. Whole cell lysates were analyzed by immunoblot analysis. Equal loading was confirmed by reprobing for β-actin. (B) Effect of BSE (1.7 mg/mL) treatment on the activation of caspase-8 as detected by immunocytochemistry. A375 cells were treated with BSE for 24 h. A representative picture from three independent experiments with similar results is shown.

Since the expression of proteins involved in the intrinsic apoptotic pathway were unaffected by BSE treatment, we speculated that BSE-induced apoptosis might be occurring through an alternative apoptotic pathway. We evaluated the involvement of the extrinsic pathway in BSE-mediated apoptotic death of A375 cells. Western blot analysis of BSE treated cells showed a significant increase in the active forms of caspase-8 in a dose-dependent fashion. In addition, caspase-7 was also upregulated in BSE treated cells (Figure 4A). Immunocytochemical studies further validated the increase in the expression of caspase-8 in BSE treated cells (Figure 4B).

### BSE treatment induced up-regulation of TRAIL/DR-4 expression in A375 melanoma cells

**Figure 5:**
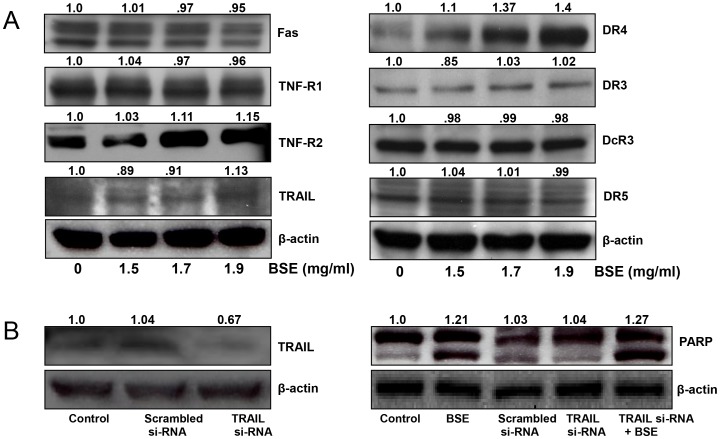
Effect of BSE treatment on expression of death domain receptor–dependent apoptotic proteins. (A) Representative immunoblots showing expression of Fas, TNF-R1, and TNF-R2, TRAIL, DR4, DR3, DcR3, and DR5 in cells treated with BSE. (B) Representative images showing expression of TRAIL protein and PARP in nonsilencing siRNA control and TRAIL-siRNA (12 µL) transfected cells with or without treatment with BSE (1.7 mg/mL) as analyzed by Western blotting. Equal loading was confirmed by reprobing for β-actin. The values above the figures represent relative density of the bands normalized to β-actin. A representative picture from three independent experiments with similar results is shown.

It has been shown that apoptosis induced through the extrinsic pathway is triggered by activation of death receptors, such as Fas, tumor necrosis factor receptor (TNF-R), Death Receptor (DR) -3, DR-4, and DR-5, by their respective ligands. DR ligands characteristically initiate signaling via receptor oligomerization, which in turn results in the recruitment of specialized adaptor proteins and activation of the caspase cascade [19]. Because activation of caspase-8 was evident, we investigated the expression of death receptors and their ligands in BSE-treated A375 melanoma cells. Western blot analysis showed that BSE treatment did not induce any change in DR-3, DcR-3, DR-5, Fas, and TNF-R1 protein expression. In sharp contrast, treatment of cells with BSE was observed to cause a dose-dependent induction of TNF-R2, TRAIL and DR-4 protein expression (Figure 5A). These data suggested that BSE-mediated apoptosis induction in A375 melanoma cells might occur through activation of the TRAIL/DR-4 apoptotic pathway.

### Effect of TRAIL siRNA on apoptosis in A375 melanoma cells

In order to verify if TRAIL/DR-4 is the mechanism by which BSE induced apoptosis in A375 cells, we examined the effect of BSE on A375 cells transfected with TRAIL siRNA, using PARP as a marker of apoptotic cell death. However, A375 cells transfected with TRAIL siRNA and treated with BSE (1.7 mg/mL) exhibited PARP cleavage demonstrating that TRAIL/DR4 is not the primary mechanism involved in the apoptotic cell death observed in BSE treated A375 cells (Figure 5B).

### BSE activated the NF-κB pathway in A375 melanoma cells

**Figure 6:**
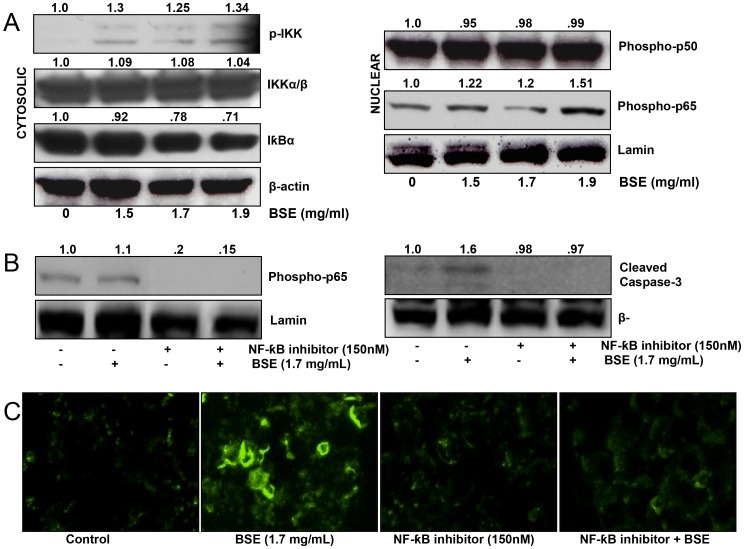
BSE treatment induced NF-κB activation associated with apoptosis in A375 melanoma cells. (A) BSE treatment (1.7 mg/mL for 24 h) induces phosphorylation of IKK and nuclear translocation of NF-κB/p65. Cytosolic and nuclear lysates were analyzed by immunoblot analysis. Equal loading was confirmed by reprobing for β-actin and lamin. (B) Inhibition of NF-κB activation by NF-κB activation inhibitor IV and subsequent inhibition of BSE induced cleavage of caspase-3. Cytosolic and nuclear lysates were analyzed by immunoblot analysis. Equal loading was confirmed by reprobing for β-actin and lamin. The values above the figures represent relative density of the bands normalized to β-actin or lamin. (C) Annexin staining showing that BSE treatment of cells pre-treated with NF-κB inhibitor IV failed to undergo apoptosis. A representative picture from three independent experiments with similar results is shown.

Tumor necrosis factor is associated with multiple functions in cancer biology, including cancer cell survival, angiogenesis, migration and invasion. These cellular responses are mediated through two distinct receptors, designated TNF-R1 and TNF-R2. In certain cell types, TNF-R1/2 signaling can initiate phosphorylation of IκB kinase (IKK) leading to nuclear translocation of NF-κB [20]. Since we observed an induction in the expression of TNF-R2 by BSE, we decided to investigate the effect of BSE on the activation of NF-κB pathway. Western blot analysis of the cytosolic fractions of BSE treated cells demonstrated increased phosphorylation of IKKα/β. Furthermore, we found that BSE treatment resulted in phosphorylation and degradation of IκBα with subsequent decrease in its cytosolic levels (Figure 6A). We then examined the nuclear fractions, which showed an increase in NF-κB/p65 levels in BSE treated cells. In contrast, no appreciable difference in NF-κB/p50 levels was detected between the treated and untreated groups. These data suggest that BSE treatment activated NF-κB signaling in A375 cells.

### Inhibition of NF-κB activation prevented induction of BSE mediated apoptosis in A375 melanoma cells

Despite a large body of data supporting the cytoprotective role of NF-κB, there is growing evidence that NF-κB can behave in a proapoptotic fashion depending on the inducing stimulus and cell context [21]. Therefore, we examined whether activation of the NF-κB pathway was involved in BSE-induced apoptosis. After establishing that pretreatment of cells with a NF-κB Activation Inhibitor IV Calbiochem (150 nM) 2 h prior to BSE exposure suppressed NF-κB activation in A375 cells, as evidenced by diminished translocation of NF-κB/p65 into the nucleus, we evaluated apoptosis in BSE treated A375 cells using cleavage of procaspase-3 and annexin V staining as apoptotic markers (Figure 6B and C). Remarkably, BSE treated cells exposed to NF-κB inhibitor did not exhibit cleavage of procaspase-3 or annexin V staining, indicating that stimulation of the NF-κB pathway is required for BSE-induced apoptosis of A375 melanoma cells.

## Discussion

This study was designed to show the chemopreventive/chemotherapeutic potential of BSE against highly aggressive metastatic human melanoma cells. It has been suggested that isoflavones in the form of aglycones exhibit higher bioavailability and bioactivity than isoflavones in other forms [22]. We obtained BSE through solid-state biofermentation of soybean using *A. awamori* fungi as a β-glucosidase producer in order to convert the glucoside isoflavones to their aglycone forms. Accordingly, we found that treatment of 451Lu and A375 human metastatic cells with BSE resulted in decreased cell growth and viability. In contrast, cell viability was not affected when cells were treated with NBSE, which signifies the importance of soybean biotransformation in conferring anti-proliferative activities against melanoma cells.

Apoptosis is a physiological process that functions as an essential mechanism of tissue homeostasis and is regarded as the preferred way to eliminate unwanted cells [23]. We observed that the primary mechanism involved in BSE-mediated inhibition of A375 melanoma cell viability was through induction of apoptosis, which was verified by fluorescence microscopy and immunoblot studies of caspases -3, -7, -8 and PARP cleavage. In this regard, it has been shown that activation of the intrinsic apoptotic pathway is characterized by increased permeability of the outer membrane of the mitochondria, with subsequent release of cytochrome c and activation of caspase-9 which, in turn, cleaves and activates caspase -3 and -7 [24]. Our results showed that caspase-9 was not activated in A375 cells treated with BSE, at least at the time point studied. Additionally, BSE had no effect on the expression of proapoptotic proteins Bid and Bax. In contrast, a clear stimulation of the death receptor pathways could be appreciated in BSE treated cells, leading us to speculate that BSE activation of the extrinsic pathway through stimulation of the death receptors may precede that of the intrinsic pathway. Further studies examining the dose response curve at increased duration of exposure may show us the involvement of the mitochondrial pathway at extended time points.

We also observed that BSE treatment increased the expression of the TRAIL receptor DR4 in melanoma cells, suggesting an amplification of the TRAIL-induced apoptotic signaling pathway. This is consistent with earlier studies that have demonstrated that soy isoflavones, daidzein, genistein and equol, augment TRAIL-induced apoptosis in LNCaP cells [19]. However, silencing studies demonstrated that TRAIL/DR4 is not the only pathway through which BSE induced apoptosis of A375 melanoma cells. Thus, the involvement of specific death receptor ligands involved in BSE induced apoptosis are being scrutinized in our ongoing studies.

An interesting feature of our study was that BSE induced phosphorylation and activation of the NF-κB pathway, which was evident from increased NF-κB/p65 nuclear translocation in BSE-treated cells. It is generally accepted that NF-κB activation is associated with cell survival [25]. However, induction of apoptosis is now being linked to activation of NF-κB in the cell, as emerging data points to a pro-apoptotic role of certain dimers of the transcription factor. Indeed, stimulation of the NF-κB pathway by specific agents has been shown to cause nuclear translocation of NF-κB/p65 complexes with subsequent repression of transcription of NF-κB-regulated antiapoptotic genes [26], [27]. Accordingly, NF-κB may have a dual role, either as an inhibitor or an activator of apoptotic cell death, depending on the levels of RelA and c-Rel proteins [25], [28]. In this context, IκBα degradation and NF-κB activation has been shown to precede cell death in cisplatin treated human head and neck squamous cell carcinoma cells [29]. Moreover, Paclitaxel/Taxol, a naturally occurring antimitotic agent, was shown to induce apoptotic cell death in two cancer cell lines via a pathway independent of mitotic arrest [30]. Authors showed that short exposures to paclitaxel could activate or regulate multiple components of the NF-κB/IκB pathway, which in turn promoted the nuclear translocation of NF-κB and its DNA binding activity. These authors further showed that inactivation of this pathway by an NF-κB inhibitor caused a block of paclitaxel-induced apoptosis.

We found that BSE induced apoptosis of A375 melanoma cells was dependent on activation of NF-κB signaling, where BSE treated cells showed activation and translocation of NF-κB/p65 subunit to the nuclei, associated with increased PARP and caspase-3 cleavage. Inhibition of signaling by treating the cells with a NF-κB inhibitor abrogated the induction of apoptosis. However, additional studies are needed to clearly understand the cross talk between the NF-κB/p65 signaling and the extrinsic apoptotic pathway. In-depth analysis of the target genes regulated by NF-κB/p65 will enable us to define the precise mechanism underlying BSE-induced cytotoxicity.

In summary, our findings shed light on the mechanistic basis of the cytotoxicity observed in BSE treated melanoma cells and suggest NF-κB/p65 mediated apoptosis through activation of the extrinsic pathways as the mechanism of cell death in these cells. These results identify an anti-cancer activity of BSE that is highly relevant to melanoma chemopreventive/chemotherapeutic potential in humans.

## Material and Methods

### Antibodies and reagents

Primary antibodies for poly(ADP-ribose) polymerase (PARP), caspase-3, caspase-7, cleaved caspase-8, caspase-9, phospho-Bad (p-Bad) (Ser112), Bid, Bax, Fas, TNF-R1, TNF-R2, DR4, and anti-mouse or anti-rabbit secondary antibody horse radish peroxidase (HRP) conjugates were purchased from Cell Signaling Technology (Beverly, MA). TRAILsiRNA and β-actin was purchased from Santa Cruz Biotechnology (Santa Cruz, CA). Amaxa Cell Line Nucleofector kit was purchased from Lonza (Lonza, Basel, Switzerland). The BCA protein assay kit and SuperSignal West Pico chemiluminescent substrate kit were purchased from Pierce (Pierce, Thermo Scientific, Rockford, IL). The mini-protean precast Tris-Glycine gels were purchased from BioRad (Hercules, CA). Anti-mouse or anti-rabbit secondary antibody biotinylated conjugates were obtained from Vector Laboratories (Burlingame, CA). Annexin-V-FLUOS staining kit was purchased from Roche Diagnostic Corporation (Indianapolis, IN). NF-κB Activation Inhibitor IV was purchased from Calbiochem (EMD Millipore Corporation, Billerica, MA).

### Biotransformed soybean extract (BSE)

Soybean seeds (*Glycine max* – Codetex 232 variety) were raised in the state of Parana (Brazil). Seeds dried at 37°C in a stove with air circulation were ground in a knife mill to fine particles (0.3 mm-mean diameter). The soybean flour was extracted with isopropanol (1∶2 parts w/w) by agitation (500 rpm) for 2 h at room temperature. Defatted soybean flour was obtained by centrifuging the suspension at 3000 g for 30 min and drying the separated residue in a water bath [12].

Fungi used in the fermentation processes were *A. awamori* (ATCC 22342). Starter micro-organisms were previously activated by transference to potato dextrose agar (PDA, Oxoid, Basingstoke,UK) slants and incubation at 30°C for 5 days. Defatted soybean flour (10 g) was dispersed in 250 ml Erlenmeyer flasks with 10 ml distilled water and autoclaved at 121°C for 15 min. Solid state fermentation was performed by evenly spraying 1.0 ml spore suspension of test organisms (10^7^ spores ml^−1^) onto the autoclaved soybean substrate. After mixing, the inoculated soybean substrate was incubated for 48 h at 30°C [12]. For the non-biotransformed soybean extract the soybean was sprayed with 1.0 ml distilled water.

Powdered samples (10 g) of unfermented and fermented soybean flour were extracted with 80% aqueous ethanol solution (1∶4 w/v) under agitation for 2 h at 25°C, and the homogenates NBSE and BSE, respectively, were vacuum filtered.

### Cell culture and treatment

A375 (ATCC, VA) and 451Lu human melanoma cell lines kindly provided by Dr. Meenhard Herlyn (Wistar Institute, PA) were cultured in DMEM and MEM media respectively (GIBCO, Carlsbad, CA), supplemented with 10% FBS (GIBCO) and 1% antibiotics (10,000 I.U.·mL^−1^ penicillin and 10,000 µg·mL^−1^ streptomycin, GIBCO), at 37°C with 5% CO_2_ in a humid environment. For dose-dependent studies, biotransformed (BSE) and non-biotransformed (NBSE) soybean extracts were dissolved in dimethyl sulfoxide (DMSO). Cells (70% confluent) were treated with soybean extracts for 24 h at 37°C with specified doses in complete growth medium, and harvested for further studies.

### Cell viability assay

The effect of NBSE and BSE soybean extracts on the viability of A375 and 451Lu cells was determined by MTT (3-[4,5-dimethylthiazol-2-yl]-2,5-diphenyl tetrazoliumbromide) assay [31]. The cells (70% confluent) were treated with NBSE and BSE (0–2.2 mg/mL for A375, and 0–1.6 mg/mL for 451Lu) in complete culture medium in 24-well microtiter plates for 24 h at 37°C and 5% CO_2_ in a humidified chamber. Twenty-four hours post-treatment, 300 µL of MTT reagent (0.5 mg/mL final concentration in medium) was added to each well and incubated for 2 h. The MTT solution was removed from the wells by aspiration and the formazan crystals were dissolved in DMSO (300 µL). Absorbance was recorded on a microplate reader (ThermoLabsystems, Fisher) at 540 nm wavelength. Vehicle (DMSO)-treated cells were regarded as 100% viable.

### Apoptosis assay

To investigate whether BSE induced apoptosis in A375 cells, we analyzed the dose dependent effect of BSE (1.5–1.9 mg/mL) on cellular morphology after 24 h treatment using the Annexin-V-FLUOS Staining kit for detection of apoptotic and necrotic cells according to vendor's protocol (Roche Diagnostics Corporation, Indianapolis, IN). This kit uses a dual-staining protocol in which the apoptotic cells are stained with Annexin-V (green fluorescence), and the necrotic cells are stained with PI (red fluorescence). Fluorescence was visualized using a Nikon Eclipse Ti system (Nikon Instruments Inc.; Tokyo, Japan), and digital images were captured with an attached CoolSNAP camera (Roper Scientific, Trenton, NJ) linked to a computer.

### Preparation of total cell lysates

Cell lysates were prepared as described previously [32]. Briefly, A375 cells treated with BSE, were washed twice in PBS (10 mM, pH 7.4) and incubated for 15 min with 0.15 mL ice-cold RIPA lysis buffer (25 mM Tris-HCl pH 7.6, 150 mM NaCl, 1% NP-40, 1% sodium deoxycholate, 0.1% SDS; Pierce Biotechnology, Rockford, IL) with freshly added protease inhibitor cocktail (Protease Inhibitor Cocktail Set III; Calbiochem, La Jolla, CA) and 0.2 mM sodium vanadate. The cells were scraped and the lysate was collected in a microcentrifuge tube, after which it was passed through a syringe to break up cell aggregates. Homogenized lysates were then centrifuged at 13 000 *g* for 25 min at 4°C to remove cell debris and the supernatant (total cell lysate) was collected and stored at −80°C. Protein concentration was determined by BCA protein assay kit according to the manufacturer's protocol (Pierce Biotechnology, Rockford, IL).

### Preparation of cytosolic and nuclear lysates

BSE treated cells were washed twice in PBS (10 mM, pH 7.4) and then incubated for 15 min in 0.3 ml ice-cold lysis buffer (10 mM HEPES pH 7.9, 10 mM KCl, 0.1 mM EDTA, 0.1 mM EGTA, 1 mM DTT, 1 mM PMSF) with freshly added protease inhibitor cocktail (Protease Inhibitor Cocktail Set III; Calbiochem, La Jolla, CA, USA) and 0.2 mM sodium vanadate. After that, 12.5 µL of 10% Nonidet P-40 was added and the contents were mixed on a vortex and then centrifuged for 1 min (13 000 *g*) at 4°C. The supernatant was stored as cytosolic lysate at −80°C. The nuclear pellet was resuspended in 40 µL of ice-cold nuclear extraction buffer (20 mM HEPES pH 7.9, 0.4 M NaCl, 1 mM EDTA, 1 mM EGTA, 1 mM DTT, 1 mM PMSF) with freshly added protease inhibitor cocktail (Protease Inhibitor Cocktail Set III; Calbiochem, La Jolla, CA, USA) and 0.2 mM sodium vanadate and incubated for 30 min with intermittent mixing. The tubes were centrifuged for 5 min (14 000 *g*) at 4°C, and the supernatant (nuclear extract) was stored at −80°C. The protein concentration was determined by the BCA protein assay kit using the manufacturer's protocol (Pierce, Rockford, IL, USA).

### Western blotting

For western blotting, 40 µg protein was resolved over 8–12% polyacrylamide gels and transferred to a nitrocellulose membrane. The blot was blocked in blocking buffer (7% non-fat dry milk, 1% Tween 20; in 20 mM Tris-buffered saline, pH 7.6) for 1 h at room temperature and incubated with the appropriate monoclonal or polyclonal primary antibody in blocking buffer for 1.5 h to overnight at 4°C, followed by 1.5 h incubation with anti-mouse or anti-rabbit secondary horseradish peroxidase conjugated antibody. The membrane was washed several times and the bound complex was detected by chemiluminescence (ECL kit, Amersham Biosciences, UK) and autoradiography using XAR-5 film obtained from Eastman Kodak Co. (Rochester, NY) or developed by digital camera using the program ChemiDoc XRS Software (Biorad, Hercules, CA).

### Immunocytochemistry

A375 cells were seeded in two-chamber tissue culture glass slides and treated with BSE (1.7 mg/mL) for 24 h. After treatment, cells were washed twice with 1X PBS and fixed in 4% paraformaldehyde in PBS for 30 min at room temperature. After three washes with 1X PBS, the cells were blocked with Background Sniper solution (Biocare Medical, Concord, CA) for 20 min and incubated with caspase-3 or caspase-8 antibody (1∶400, and 1∶100, respectively, in 2% bovine serum albumin and 2.5% goat serum in 1X PBS) overnight at 4°C. Then, the cells were washed and incubated with biotin-conjugated secondary antibody (1∶500 in the same antibody dilution buffer) for 2 h at room temperature in the dark. Cells were then treated with ABC Conjugate (R.T.U. Vectastain Kit, Burlingame, CA) for 30 min and incubated with 3,30-diaminobenzidine (Dako Liquid DAB + Substrate Chromogen System, Dako Corp,CA) until desired stain intensity developed. Cells were counterstained with 10% hematoxylin (Vector Hematoxylin Qs Nuclear Counsterstain,Burlingame, CA) in water, dehydrated through graded ethanol series, cleared in xylene and mounted. Staining was visualized using a Nikon Eclipse Ti inverted microscope.

### Statistical analysis

Results were expressed as the means ± SD. Statistical analysis between controls and treatments were performed by Student's t-test, where a P≤0.05 was considered statistically significant.
